# Unlocking the Potential of Negative Pressure Wound Therapy in Diabetic Foot Ulcers: A Systematic Review and Meta-Analysis

**DOI:** 10.7759/cureus.96474

**Published:** 2025-11-10

**Authors:** Roshan A Kadavan, Nikhil Deans, Rithin Punnackal Joseph, Jisna Vincent, Ajanth S, Merary Merybal

**Affiliations:** 1 General Medicine, Be Well Group of Hospitals, Chennai, IND; 2 General Medicine, Clinician Care Hospital, Chennai, IND; 3 Medicine and Surgery, Indian Council of Medical Research, Kerala, IND; 4 Medicine and Surgery, Indian Spinal Injuries Center, Kerala, IND; 5 Anaesthesiology, Clinician Care Hospital, Chennai, IND

**Keywords:** dfu (diabetic foot ulcer), foot ulcer, negative pressure wound therapy (npwt), npwt, vac, wound healing

## Abstract

Diabetes mellitus (DM), which affects millions globally, is rising exponentially. Diabetic foot ulcers (DFUs) are a major health and economic concern in patients with uncontrolled DM. Complex factors such as neuropathy, ischemia, and susceptibility to infection contribute to the development of DFUs, which can lead to amputations and significant mortality. Traditional DFU therapies often struggle to address the diversity and complexity of ulcers. Current guidelines for DFU care recommend Negative Pressure Wound Therapy (NPWT) as a potential treatment option. NPWT applies controlled negative pressure to optimize wound healing. Despite its promise, limited evidence, varying methodologies, and restricted data accessibility necessitate a comprehensive evaluation.

Our study adhered to PRISMA standards and conducted an extensive search across multiple databases, including PubMed, Embase, and the Cochrane Library, to identify randomized controlled trials (RCTs) published after 2008. The search included targeted keywords related to diabetic foot complications and NPWT to evaluate the safety and efficacy of this approach compared with conventional wound treatments.

In the meta-analysis comprising 11 studies and a total of 1,135 participants, significant outcomes favored NPWT for DFUs. NPWT demonstrated a substantial impact on complete wound closure, with an OR of 2.193 (95% CI: 1.562-3.079; p < 0.0001). Ulcer healing also showed a statistically significant improvement, with an OR of 2.771 (95% CI: 1.511-5.082; p = 0.0010). Granulation tissue development improved markedly, as indicated by a standardized mean difference of -1.3384 (95% CI: -1.5577 to -1.1192; p < 0.0001). NPWT notably reduced amputation rates by 63%, with an OR of 0.368 (95% CI: 0.182-0.746). Although there was a trend toward fewer adverse events with NPWT, the effect did not reach statistical significance (log OR: 0.1548; 95% CI: -0.4364 to 0.7460).

These findings underscore the clinical efficacy of NPWT in the management of DFUs. In conclusion, this systematic review and meta-analysis confirm the effectiveness of NPWT in improving wound closure, accelerating recovery, and reducing amputation rates in patients with DFUs.

## Introduction and background

Diabetes mellitus (DM) has emerged as a global health concern of unprecedented proportions, affecting millions worldwide and imposing a substantial burden on healthcare systems [1,2]. It is broadly classified into two main types: type 1 DM, caused by autoimmune destruction of pancreatic β-cells leading to insulin deficiency, and type 2 DM, characterized by insulin resistance and impaired insulin secretion. Globally, over 463 million people were living with diabetes in 2019, representing approximately 9.3% of the adult population. This figure is projected to rise to 10.9% by 2045 [3,4], making it one of the most significant health concerns on a global scale. The prevalence continues to increase worldwide, including in countries such as the United Kingdom, the United States, and India, where diabetes poses a major public health challenge [5,6]. Chronic hyperglycaemia in diabetes leads to numerous microvascular and macrovascular complications. Among these, diabetic foot ulcers (DFUs) represent one of the most serious and debilitating outcomes, distinguished by their alarming frequency, complex pathophysiology, and substantial economic impact [7]. DFUs are defined as exposed lesions or injuries on the lower extremities of individuals with diabetes [8].

The emergence of these ulcers can be attributed to a confluence of factors, including neuropathy, ischaemia, and susceptibility to infections [9]. These factors diminish sensory perception and compromise regenerative capacity, allowing minor injuries to progress into ulcers characterized by delayed healing and increased risk of complications [10,11]. DFUs can lead to severe infections, tissue damage, and, in certain cases, may necessitate amputation of the affected limb [12]. This condition affects a significant proportion of individuals with diabetes, potentially impacting approximately one-third of them during their lifetime [13]. The potential progression of these ulcers is particularly alarming, leading to severe consequences such as major limb amputations and high mortality rates. A study on the mortality burden of DFUs revealed distinct five-year mortality rates among different ulcer types: ischemic ulcers demonstrated a mortality rate of 55%, neuropathic ulcers 45%, and neuroischemic ulcers 18% [14]. Moreover, diabetic patients who undergo major lower limb amputations face a distressing 50% mortality rate within five years [15].

The implications of this reality are profound. The ramifications of DFUs are extensive, encompassing reduced physical functionality, poor quality of life, and escalating healthcare costs. Although specialized multidisciplinary treatment has shown promise in facilitating recovery in many cases, a considerable number of patients do not respond adequately to conservative interventions or experience recurrence, with rates reaching as high as 30% after one year and 70% after 10 years [16]. The research and implementation of advanced management strategies, coupled with prompt medical intervention, play a pivotal role in mitigating the progression of these ulcers and reducing their potential impact on an individual’s overall health and well-being.

Conventional therapies encounter difficulties when it comes to the selection of dressings for DFUs [17,18]. The heterogeneous characteristics of DFUs, which can arise from multiple etiological factors and affect distinct anatomical sites, present a significant obstacle to the implementation of a universally applicable dressing selection strategy. This diversity underscores the necessity of adopting alternative advanced approaches to wound treatment [17]. Within the realm of managing DFUs, Negative Pressure Wound Therapy (NPWT), also known as vacuum-assisted closure (VAC), has emerged as a noteworthy therapeutic alternative, offering potential advantages in facilitating wound healing and preventing complications. NPWT is a recommended therapeutic modality according to National Institute for Health and Care Excellence (NICE) guidelines [19] for the management of DFUs and is commonly employed as a preliminary intervention before surgical procedures are considered.

NPWT involves the controlled application of negative pressure to the wound site using a specially designed device [20]. The level of pressure applied during NPWT may vary depending on several factors, including the specific characteristics of the wound, the type of device used, and the clinical judgment of the healthcare professional [21]. Generally, the pressure parameters for NPWT range from -50 mmHg to -125 mmHg, with most cases using pressures between -75 mmHg and -125 mmHg [22]. This technique establishes a favorable environment for wound healing through two primary mechanisms: macro-deformation and micro-deformation. Macro-deformation occurs when an open-pore foam draws the wound margins closer together, while micro-deformation involves stretching of the cells, thereby promoting cellular proliferation. The process also facilitates fluid extraction, reduces edema, maintains a thermally conducive environment, and promotes granulation tissue formation. Furthermore, secondary effects include increased cellular division, modulation of inflammatory responses, alterations in neuropeptide levels, and variable effects on bacterial populations [23-27]. The multifaceted impact of NPWT highlights its significance in promoting wound healing and tissue regeneration, enhancing blood circulation, and enabling the removal of infectious materials and exudates [22], thereby expediting the healing process.

Existing evidence suggests that NPWT can accelerate wound healing and reduce amputation rates [22]. However, the current literature is limited by methodological inconsistencies, small sample sizes, and variable outcome measures. Several trials have been terminated prematurely or remain unpublished, raising concerns about data completeness and transparency, as reported in a previous investigation by Peinemann F et al. [28]. Although newer studies have expanded the evidence base, uncertainties persist regarding optimal patient selection, treatment duration, and long-term efficacy.

Despite its promise, inconsistencies in study design and reporting have created uncertainty about NPWT’s true efficacy, underscoring the need for a systematic review. Therefore, this systematic review and meta-analysis aim to provide a comprehensive and unbiased evaluation of the efficacy and safety of NPWT in managing DFUs. Specifically, it assesses the effects on wound healing, ulcer closure, amputation rates, and adverse events, addressing current knowledge gaps and supporting evidence-based clinical practice.

## Review

Methodology

Search Strategy and Study Selection

**Figure 1:**
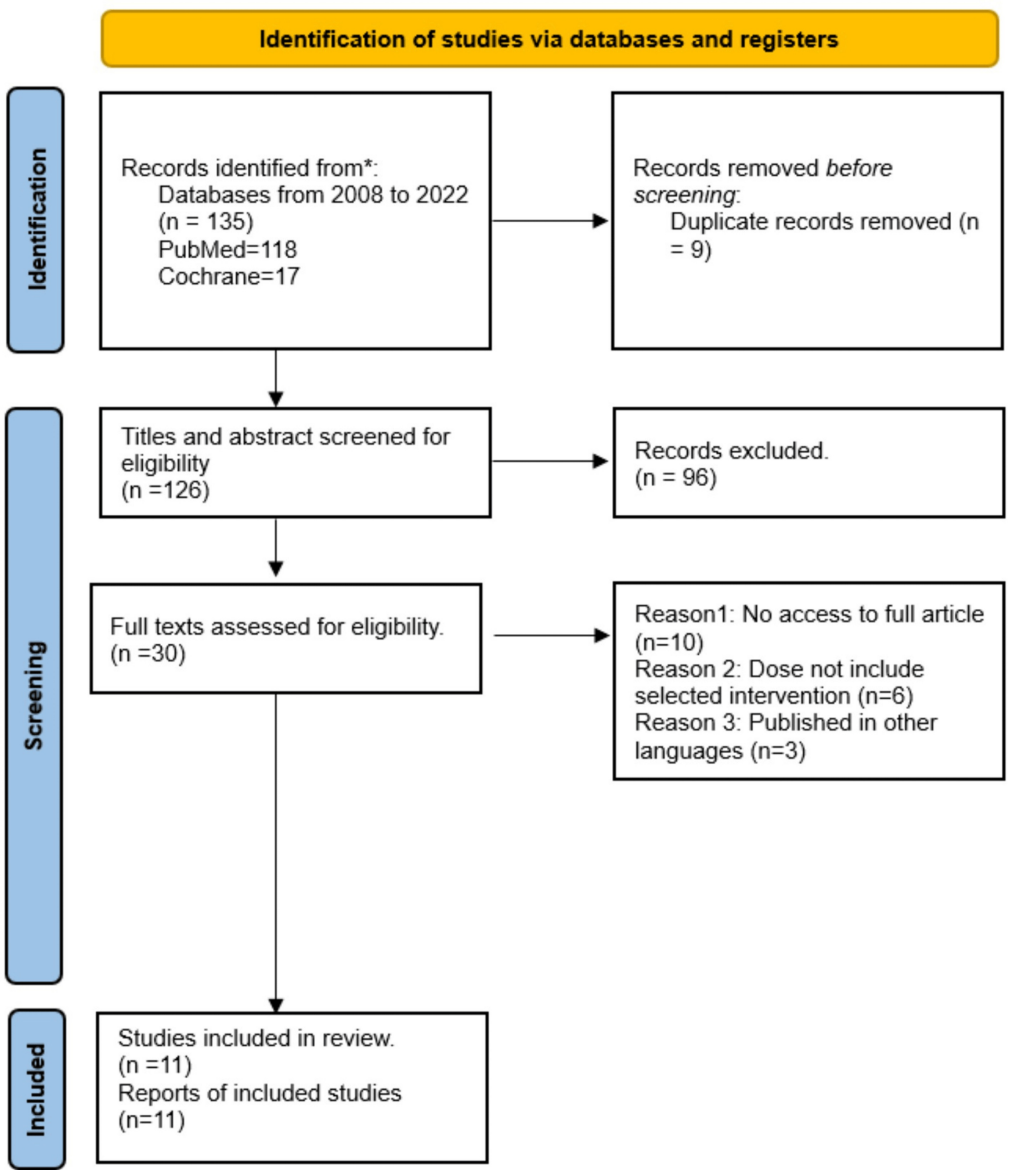
PRISMA flow diagram showing the selection of potential articles. PRISMA: Preferred Reporting Items for Systematic Reviews and Meta-Analyses.

This systematic review and meta-analysis followed the recommendations specified in the Preferred Reporting Items for Systematic Reviews and Meta-Analyses (PRISMA) checklist (Figure 1), which ensured the implementation of a comprehensive and methodologically sound research process [29].

To identify relevant randomized controlled trials (RCTs) examining the safety and effectiveness of NPWT in various clinical settings, a thorough search was conducted across several databases, including PubMed, Embase, and the Cochrane Library. The search strategy employed in this study utilized a predetermined list of keywords such as “diabetic foot,” “diabetic feet,” “foot ulcer, diabetic,” “foot, diabetic,” “negative pressure wound therapy,” “vacuum assisted closure,” “topical negative pressure therapy,” “negative pressure dressings,” “VAC,” “NPWT,” and relevant Boolean search strings for each database (Table 1). The objective of the search was to identify RCTs published between January 2008 and June 2022. The primary focus was on individuals diagnosed with diabetic foot conditions who received NPWT interventions, compared with those treated using conventional wound therapies. Disagreements regarding data inclusion or extraction were resolved by consensus, with input from a statistician when required.

**Table 1:** Search strings used in each electronic database. MeSH Terms: Medical Subject Heading terms; VAC: Vacuum-Assisted Closure; NPWT: Negative Pressure Wound Therapy.

Electronic Database	Search Strings
PubMed	("diabetic foot"[MeSH Terms] OR "diabetic foot"[Title/Abstract] OR "diabetic feet"[Title/Abstract] OR "foot ulcer, diabetic"[Title/Abstract] OR "foot, diabetic"[Title/Abstract]) AND ("negative pressure wound therapy"[MeSH Terms] OR "negative pressure wound therapy"[Title/Abstract] OR "vacuum assisted closure"[Title/Abstract] OR "topical negative pressure therapy"[Title/Abstract] OR "negative pressure dressing"[Title/Abstract] OR "VAC"[Title/Abstract] OR "NPWT"[Title/Abstract]) AND ("randomized controlled trial"[Publication Type] OR "randomized"[Title/Abstract] OR "randomised"[Title/Abstract]) Filters: Humans, English language, publication date from 2008 to 2022.
Cochrane	("diabetic foot" OR "diabetic feet" OR "diabetic foot ulcer") AND ("negative pressure wound therapy" OR "vacuum assisted closure" OR "topical negative pressure therapy" OR "negative pressure dressing" OR "VAC" OR "NPWT") In Trials. Filters: Randomized controlled trials (RCTs), publication years 2008-2022.
Embase	('diabetic foot'/exp OR 'diabetic foot':ti,ab OR 'diabetic feet':ti,ab OR 'diabetic foot ulcer':ti,ab) AND ('negative pressure wound therapy'/exp OR 'vacuum assisted closure':ti,ab OR 'topical negative pressure therapy':ti,ab OR 'negative pressure dressing':ti,ab OR 'VAC':ti,ab OR 'NPWT':ti,ab) AND ('randomized controlled trial'/exp OR 'randomized':ti,ab) Filters: Human studies, English language, publication years 2008-2022.

Study Selection Criteria

The literature considered for inclusion comprised RCTs. Eligible studies were those published after January 2008 and up to June 2022.

Study Population

Individuals diagnosed with diabetic foot problems, including foot lesions of various aetiologies (e.g., neuropathy, ischaemia, or neuroischaemia) associated with diabetes, were included. Studies comparing NPWT with standard dressing techniques, such as saline dressings or conventional moist gauze, were eligible. Research articles published in English or other languages with available translations for data extraction were included, provided the study population consisted of diabetic adults aged 18 years or older. The study incorporated several outcome indicators, including wound healing rate, time to wound healing, adverse event occurrence, amputation rate, mortality, recurrence rate, and other relevant measures.

Studies were excluded if they were non-randomized, did not clearly describe the NPWT intervention methodology, had a cumulative sample size of fewer than 10 participants, lacked a control or comparison arm, were unpublished or non-peer-reviewed, were written in languages without adequate translation, or lacked sufficient outcome measures or methodological consistency. Studies primarily focusing on wound etiologies other than DFUs were also excluded.

Data Collection

Following the elimination of duplicate articles, the screening process consisted of an initial examination of the titles and abstracts, followed by a detailed evaluation of the full-text articles in accordance with the established inclusion and exclusion criteria. Two studies, Armstrong DG et al. (2005) and Blume PA et al. (2008), preceded the stipulated timeframe in our criteria. These studies were intentionally included because they represent the first major RCTs evaluating NPWT in DFUs. Their inclusion provides essential historical and methodological context, especially since few high-quality RCTs were available during the early stages of NPWT research, and these studies helped shape both clinical practice and subsequent investigations in this area. Data collected during the full-text review included various parameters such as author information, publication date, details of the intervention and control groups, patient characteristics, and key clinical outcomes.

Data Analysis

To conduct an in-depth analysis of the studies incorporated into this meta-analysis, data synthesis and statistical analyses were performed using the Jamovi software. Binary data were synthesized using Odds Ratios (OR) with 95% CI, while continuous data were synthesized using Standardized Mean Differences (SMD), particularly for granulation tissue outcomes, to account for variations in measurement scales across studies (e.g., percentage granulation area, mean time to ≥90% granulation). The I² statistic and the Q-test were the primary methods used to evaluate the degree of heterogeneity among the selected studies. The threshold for significant heterogeneity was set at I² ≥ 25% or p ≤ 0.1. The degree of heterogeneity determined whether a random-effects model or a fixed-effects model was more appropriate for analysis. For the outcome of complete ulcer closure, statistical heterogeneity was negligible; however, clinical heterogeneity existed among the included studies, particularly regarding patient demographics, wound characteristics, NPWT device types, suction pressures, treatment durations, and definitions of complete ulcer closure. Therefore, a random-effects model was applied to account for this underlying clinical variability. The random-effects model provides a more conservative pooled estimate and broader CI, which better reflects the real-world diversity of the included trials and enhances the external validity of the findings. For the remaining outcomes, ulcer healing, granulation tissue formation, amputation, and adverse events, fixed-effects models were applied, as both statistical heterogeneity (I² = 0%) and clinical heterogeneity were minimal, and the study methodologies were closely aligned.

The robustness of the findings was assessed through sensitivity analysis using the leave-one-out method. Furthermore, a funnel plot was utilized to examine the potential presence of publication bias by visualizing the distribution of study results and identifying any asymmetry that might suggest such bias. Additional sensitivity testing was conducted using the model comparison technique, wherein the outcomes derived from fixed-effects and random-effects models were thoroughly compared. This approach enabled us to test the durability and consistency of our findings by evaluating the impact of different model settings on overall outcomes. Through these comparisons, we were able to confirm the consistency of our conclusions irrespective of the statistical model employed, thereby ensuring the reliability of our meta-analysis results.

Quality Assessment of the Included Studies

The methodological quality of the included RCTs was assessed using the Jadad scale, which evaluates three key criteria: randomization (0-2 points), blinding (0-2 points), and reporting of withdrawals/dropouts (0-1 point), for a maximum score of 5.

Across the eleven studies, the overall methodological quality was rated as moderate to high. The highest-quality studies were the large multicenter RCTs, Blume PA et al. (2008) and Armstrong DG et al. (2005), which clearly described randomization procedures and follow-up, achieving scores of 4 and 3, respectively. Furthermore, an inverse relationship was observed between study size and Jadad score: larger trials with comprehensive outcomes and diverse populations exhibited higher methodological quality, whereas smaller studies with limited sample sizes or restricted endpoints demonstrated lower quality.

Despite these limitations, the direction of treatment effect remained consistent across all included studies. The higher-quality, larger-scale RCTs contributed substantially to the robustness and reliability of the pooled conclusions.

Results

Our database searches yielded 52 articles. After removing duplicates and screening titles and abstracts, we carefully reviewed the full text of these papers for eligibility. We excluded 41 papers for not meeting the inclusion criteria, such as being in a foreign language, case reports or review articles, lacking complete open access, not being RCTs, or not primarily focusing on diabetic foot wounds. Following this rigorous screening process, 11 papers were included in the meta-analysis (Table 2) [30-40]. Across the included studies, the number of participants ranged from 22 to 345, with a mean age of 56.13 years. Intervention durations varied from 7 to 112 days, and post-intervention follow-up periods ranged from 1 to 12 months. This variation in study design and duration provided a comprehensive dataset for analysis.

**Table 2:** Patient and study characteristics. NPWT: Negative Pressure Wound Therapy; N/A: Not Available; VAC: Vacuum-Assisted Closure.

Author and Year	No. of Patients	Mean Age	Treatment	Control	Duration	Primary Outcome	Secondary Outcome	Features of NPWT
Vaidhya N et al. (2015) [37]	60	56.5 / 57.3	NPWT	Standard dressing	1 week	Cost-effectiveness	Granulation tissue, wound healing, complete ulcer closure	80-120 mmHg; applied every 2-3 days
Borys S et al. (2018) [32]	75	65.4 ± 8.6 / 64.2 ± 6.8	NPWT	Standard dressing	9 days	Complete ulcer closure	Amputation rates, adverse events	120 mmHg; dressing every 2 days
Chiang N et al. (2017) [33]	22	62.0 ± 13.9 / 61.0 ± 12.9	NPWT	Standard dressing	2 weeks	Wound healing	Amputation rates	125 mmHg (first 24 hrs); dressing every 2 days
Nain PS et al. (2011) [38]	30	61.33 ± 7.63 / 55.40 ± 11.54	NPWT	Standard dressing	2 weeks	Complete ulcer closure	Wound healing, amputation rates	50-125 mmHg; applied 3 times per day
Ravari H et al. (2013) [36]	23	N/A	NPWT	Standard dressing	1 week	Complete ulcer closure	Wound healing, recurrence	125 mmHg; dressing every 3 days
Lone AM et al. (2014) [35]	56	53.8 ± 5.5 / 54.6 ± 4.8	NPWT	Saline dressing	2 months	Wound healing	Amputation rates	80-125 mmHg; dressing every 2 days
James SM et al. (2019) [34]	54	55.9 / 52.9	NPWT	Standard dressing	N/A	Granulation tissue	Amputation rates	125 mmHg; dressing every 2 days
Anjum W et al. (2022) [40]	40	N/A	NPWT	Standard dressing	6 months	Complete ulcer closure	N/A	80-125 mmHg
Shukr I et al. (2015) [39]	278	56.83 ± 11.3 / 55.88 ± 10.97	NPWT	Placebo	1 week	Granulation tissue	N/A	125 mmHg; dressing every 3 days
Armstrong DG et al. (2005) [30]	162	57.2 ± 13.4 / 60.1 ± 12.2	VAC	Placebo	3 months	Complete ulcer closure	Amputation rates, adverse events	75-125 mmHg; applied every 48 hrs
Blume PA et al. (2008) [31]	335	58 ± 12 / 59 ± 12	VAC	Placebo	3 months	Complete ulcer closure	Amputation rates, adverse events	50-200 mmHg (varied)

Complete Ulcer Closure 

**Figure 2:**
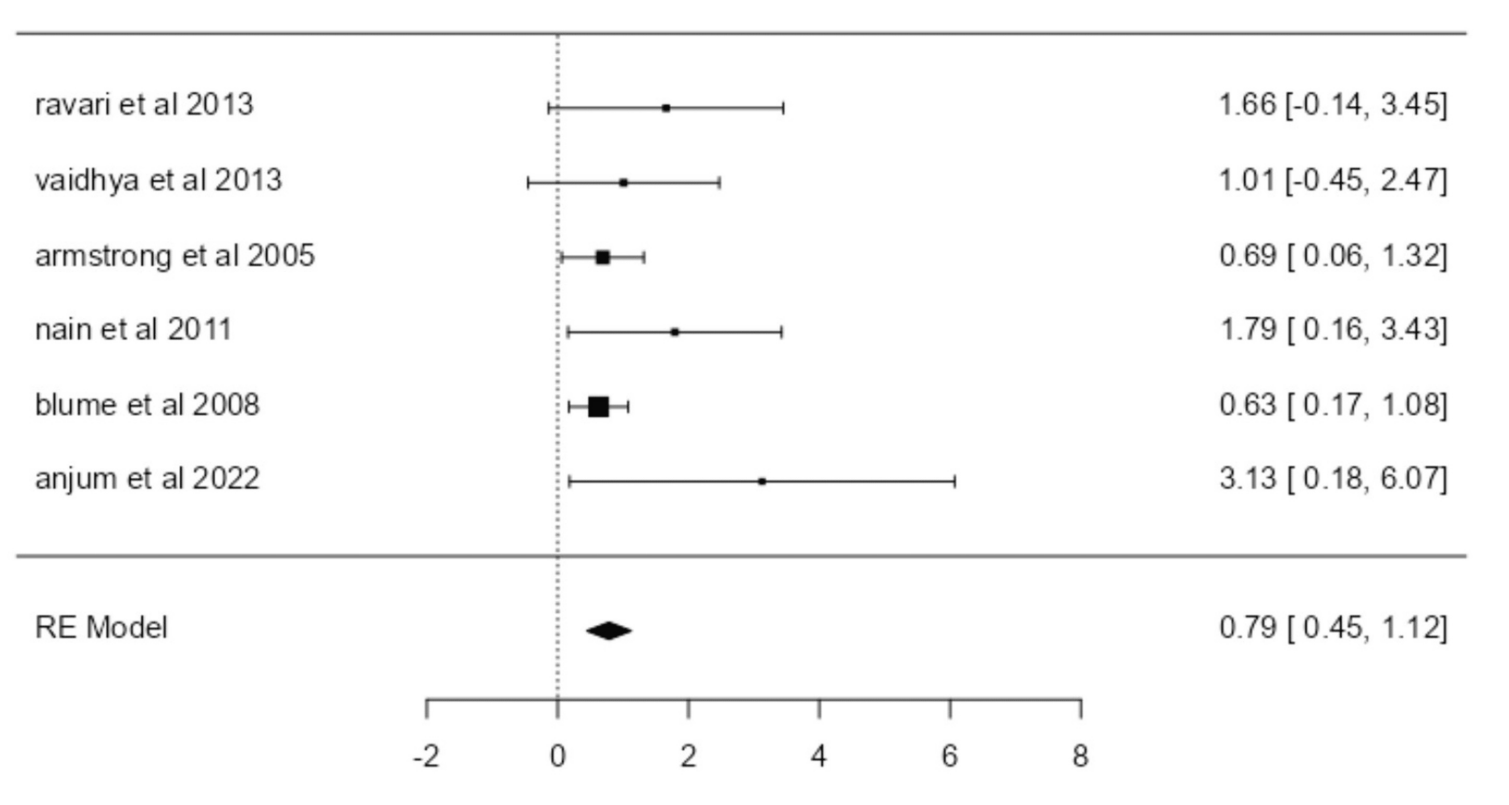
Forest plot for complete ulcer closure. Forest plot for complete ulcer closure comparing studies by Ravari H et al. (2013) [36], Vaidhya N et al. (2015) [37], Armstrong DG et al. (2005) [30], Nain PS et al. (2011) [38], Blume PA et al. (2008) [31], and Anjum W et al. (2022) [40]. RE: Random effects.

Data from six studies [30, 31, 36-38, 40] were analyzed to evaluate the efficacy of NPWT in achieving complete ulcer closure. A random-effects model was applied to account for underlying clinical heterogeneity among the included studies. The pooled log odds ratio was 0.7854 (95% CI: 0.4461-1.1246; p < 0.0001), corresponding to an odds ratio of 2.193 (95% CI: 1.562-3.079) (Figure 2). Heterogeneity was not significant (Q = 1.4873, p = 0.8289; I² = 0.0000%). Although Blume PA et al. [30] emerged as influential based on studentized residuals, no substantial outliers were identified. Funnel plot analyses indicated asymmetry (rank correlation p = 0.0167; regression p = 0.0265).

Ulcer Healing

**Figure 3:**
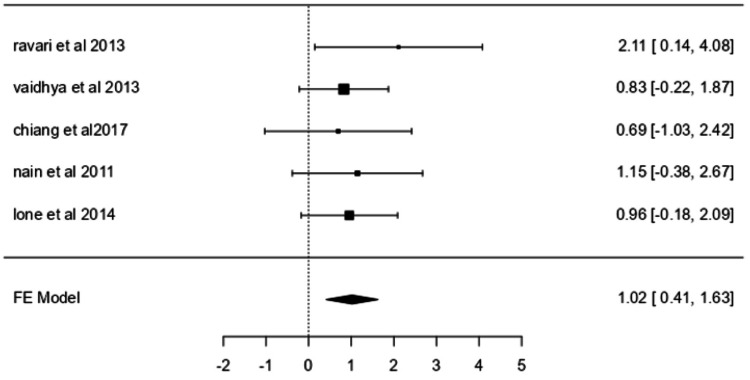
Forest plot for ulcer healing. Forest plot for ulcer healing comparing studies by Ravari H et al. (2013) [36], Vaidhya N et al. (2015) [37], Chiang N et al. (2017) [33], Nain PS et al. (2011) [38], and Lone AM et al. (2014) [35]. FE: Fixed effects.

A fixed-effects model was applied to assess ulcer healing across five studies [33, 35-38]. The analysis produced a mean log odds ratio of 1.0192 (95% CI: 0.4127-1.6258; p = 0.0010) (Figure 3), equivalent to an odds ratio of 2.771 (95% CI: 1.511-5.082). No significant heterogeneity was observed (I² = 0.0000%), and no outliers or funnel plot asymmetry were detected.

Granulation Tissue 

**Figure 4:**
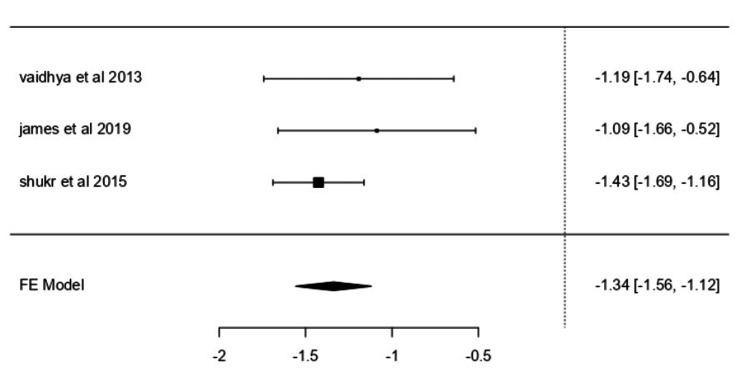
Forest plot for the complete rate of granulation tissue development. Forest plot for the complete rate of granulation tissue development comparing studies by Vaidhya N et al. (2015) [37], James SM et al. (2019) [34], and Shukr I et al. (2015) [39]. FE: Fixed effects.

Granulation tissue outcomes were reported in three trials [34, 37, 39]. Using a fixed-effects model and SMD, the pooled SMD was -1.3384 (95% CI: -1.5577 to -1.1192; z = -11.9657, p < 0.0001) (Figure 4). All individual study estimates were negative and within a narrow range (-1.4252 to -1.0877). Heterogeneity was negligible (I² = 0.0000%), and no funnel plot asymmetry was detected (rank correlation p = 0.3333; regression p = 0.2389).

Quantitative data from individual trials supported the pooled findings: Vaidhya N et al. [37] reported NPWT patients reaching ≥90% granulation in 18.8 ± 6 days, while Nain PS et al. [38] observed 75% granulation development by week two among NPWT patients. Armstrong DG et al. [30] noted that 76-100% granulation tissue was achieved earlier in NPWT-treated ulcers compared with standard therapy.

Amputation

**Figure 5:**
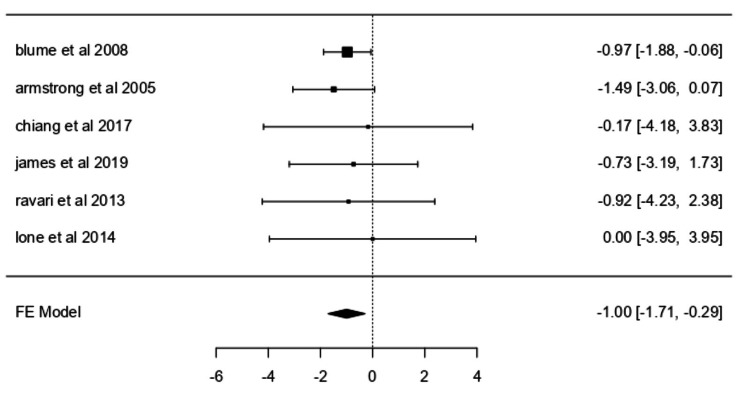
Forest plot for amputation rates. Forest plot for amputation rates comparing studies by Blume PA et al. (2008) [31], Armstrong DG et al. (2005) [30], Chiang N et al. (2017) [33], James SM et al. (2019) [34], Ravari H et al. (2013) [36], and Lone AM et al. (2014) [35]. FE: Fixed effects.

Six studies [30, 31, 33-36] evaluated amputation rates using a fixed-effects model. NPWT significantly reduced the likelihood of amputation, with a weighted log odds ratio of -0.9993 (z = -2.7729; p = 0.0056) (Figure 5), corresponding to an odds ratio of 0.368 (95% CI: 0.182-0.746). The Q-test (p = 0.9745) and I² = 0.0000% confirmed minimal heterogeneity. Although Armstrong DG et al. [30] was marginally influential, overall consistency across studies was maintained. Funnel plot analyses indicated no publication bias (rank correlation p = 0.2722; regression p = 0.6397).

Adverse Events

**Figure 6:**
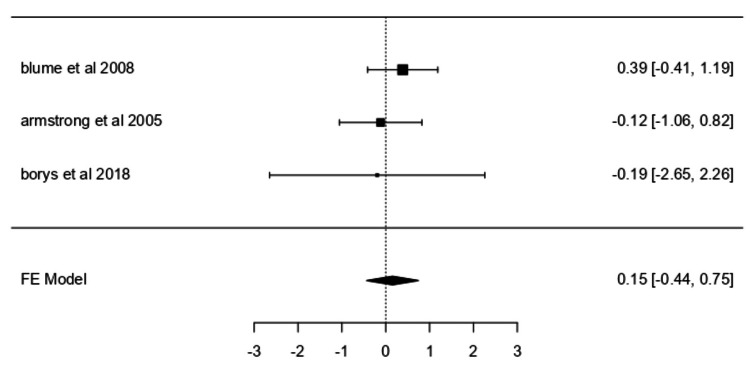
Forest plot for adverse events. Forest plot for adverse events comparing studies by Blume PA et al. (2008) [31], Armstrong DG et al. (2005) [30], and Borys S et al. (2018) [32]. FE: Fixed effects.

A fixed-effects model was used to pool data from three studies [30-32] that reported adverse event rates associated with NPWT. The pooled log odds ratio was 0.1548 (95% CI: -0.4364 to 0.7460) (Figure 6), which was not statistically significant (z = 0.5133; p = 0.6078). Heterogeneity was absent (I² = 0.0000%), and no outliers or publication bias were detected. The odds ratio ranged from 0.646 to 2.109.

Sensitivity Analysis and Publication Bias

**Figure 7:**
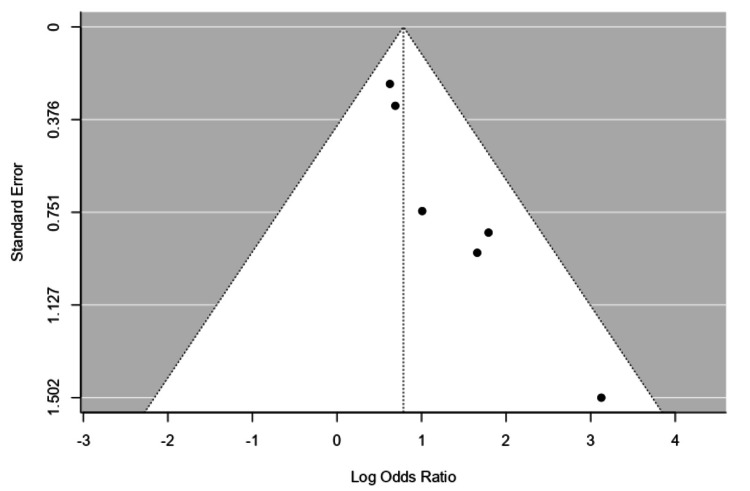
Funnel plot for complete ulcer closure. Funnel plot for complete ulcer closure comparing studies by Ravari H et al. (2013) [36], Vaidhya N et al. (2015) [37], Armstrong DG et al. (2005) [30], Nain PS et al. (2011) [38], Blume PA et al. (2008) [31], and Anjum W et al. (2022) [40].

Given the absence of significant heterogeneity, formal heterogeneity testing was deemed unnecessary. Sensitivity analyses comparing fixed-effects and random-effects models produced consistent results, confirming the robustness of the findings. No location bias was observed. However, for the outcome of complete ulcer closure, funnel plot asymmetry (Figure 7) suggested the potential presence of publication bias, warranting cautious interpretation of those specific results.

Discussion

Efficacy

This meta-analysis provides robust evidence that NPWT is a superior treatment to standard dressings for DFUs. The significant improvement in complete ulcer closure and healing rates aligns with previous research and can be attributed to NPWT’s multifactorial mechanisms, as described in the introduction. These mechanisms include enhanced perfusion, reduced edema, micro-deformation, and stimulation of cellular proliferation, collectively promoting faster and more effective healing [41,42].

Individual study findings consistently demonstrated faster granulation tissue formation with NPWT compared to standard care. Nain PS et al. [38], Vaidhya N et al. [37], and Armstrong DG et al. [30] each reported shorter timeframes and higher proportions of wounds achieving advanced granulation under NPWT, with markedly accelerated progression during the first four weeks of therapy. These individual study findings complement the pooled meta-analytic effect size (SMD = -1.3384), highlighting consistent improvements in granulation quality and rate across studies. This observation reinforces the biological plausibility of NPWT in creating a microenvironment conducive to wound repair, reflecting its capacity to enhance wound-bed maturation and healing dynamics. The emphasis on granulation tissue as a clinically meaningful endpoint, highlighted by the European Wound Management Association (EWMA), underscores its importance in evaluating the success of wound therapies. The recognition of granulation tissue formation as a key marker of wound healing further supports the conclusion that NPWT provides an optimal environment for tissue regeneration. Moreover, the variation in pressure levels across studies (ranging from -75 to -125 mmHg) may reflect the adaptability of NPWT to different wound characteristics. Interestingly, some studies [43] have suggested that pressures around -80 mmHg may produce more favorable blood flow dynamics than the conventional -125 mmHg, emphasizing the importance of individualized therapeutic adjustments.

Safety

NPWT is widely recognized in anecdotal accounts as a highly successful therapy for various types of wounds. This meta-analysis offers valuable insights by providing a comparative assessment of treatment-related adverse events between NPWT and routine dressing changes. The finding that NPWT did not show a substantial difference in adverse events compared with conventional therapy provides reassurance.

Although there is considerable literature supporting the safety and efficacy of NPWT, it is important to note the limited availability of comparative research, particularly randomized clinical trials. Therefore, the existing body of evidence substantiating NPWT’s effectiveness is not as comprehensive as its widespread clinical use might suggest. In this context, the establishment of consensus among medical experts assumes great importance to guide recommendations on the optimal use of NPWT.

Possible adverse effects linked to NPWT include infection resulting from inadequate dressing changes or wound care, minor bleeding [44] due to the impact of negative pressure on blood clots, and skin irritation or allergic reactions triggered by adhesive materials. Excessive application of negative pressure may also induce skin necrosis or tissue injury [45], causing pain and discomfort for the patient. In rare instances, perforation of adjacent tissues or organs may occur due to the applied pressure [46]. Additional complications include hematoma formation, delayed wound healing, and dressing malfunction.

Over recent years, the U.S. FDA has received numerous reports of complications and severe adverse effects associated with the use of NPWT devices [47]. In November 2009, as a direct result of these investigations, the FDA issued specific guidelines on the use of NPWT [48]. However, these reports do not necessarily indicate that NPWT is inherently unsafe, as most of the issues reported to the FDA occurred in home-care settings [45]. The absence of major complications in the RCTs included in our meta-analysis likely reflects the fact that interventions were conducted in hospitals or wound care centers, where healthcare professionals are well-versed in NPWT application, potential side effects, and appropriate management strategies following established guidelines [49].

Future studies should broaden the research scope to include ambulatory care settings. To ensure the safe and effective use of NPWT, it is imperative to conduct a thorough wound assessment, apply meticulous technique, select appropriate dressings, and continuously monitor patient progress.

Cost Effectiveness

Besides evaluating clinical outcomes, it is crucial to consider the economic implications of treatment modalities when assessing their viability and advantages [50]. DFUs are linked to significant financial repercussions due to extended hospitalizations and frequent dressing modifications, with average costs for treating simple ulcers around $9,000, contaminated DFUs at $17,000, and cases progressing to amputation reaching $45,000 [7]. Therefore, it is essential to assess the cost-effectiveness of therapies such as NPWT. Vaidhya N et al. [37] conducted a study in which 60 patients were given either a traditional bandage or NPWT. The NPWT group achieved target healing 17.2 days earlier than the control group (34.9 days), required fewer dressing changes (7.46 vs. 69.8), and showed a 90% success rate compared to 76.6% in the control group, demonstrating quicker and more cost-effective healing. Alipour [51] conducted a study in Iran to estimate the incremental cost-effectiveness ratio (ICER) of NPWT compared to Traditional Wound Care (TWC) for DFUs. The investigation assessed multiple clinical factors, including healthy, contaminated, closed, post-amputation, and mortality outcomes over a duration of one year, with treatment sessions provided monthly. The findings indicated that NPWT was associated with a reduced annual cost per patient compared with TWC ($5,165 ± $3,258 vs. $9,833 ± $5,861). Additionally, NPWT exhibited greater average effectiveness in terms of Quality-Adjusted Life Years (QALYs) (8.9026 ± 1.7622 QALYs vs. 8.7974 ± 1.855 QALYs), supported by a calculated ICER of -$44,370 per QALY. The method of application and the type of device also play a role in cost-effectiveness, though limited studies have examined the various NPWT systems in this regard. A separate investigation [52] conducted in the United States focused on assessing the cost-effectiveness of single-use Negative Pressure Wound Therapy (sNPWT) versus traditional NPWT (tNPWT) for venous leg ulcers (VLUs) and DFUs. The study compared treatment costs and ulcer-free weeks over 12 and 26 weeks, finding that sNPWT was less expensive and reduced wound contamination time compared with tNPWT. Probabilistic analysis showed that sNPWT outperformed tNPWT in 99.8% of 26-week simulations [52]. The analysis underscores the importance of incorporating cost-effectiveness into healthcare decision-making. The findings suggest that NPWT may be a promising alternative for managing DFUs, as it has the potential to yield positive clinical outcomes and demonstrate long-term economic advantages. This issue holds particular relevance within the healthcare sector, where treatment decisions are heavily influenced by budgetary constraints and economic considerations.

Limits

Only a few studies used a blinded technique, which might introduce publication bias. None of the reviewed studies examined plantar pressures [53] in diabetics at risk of foot ulcers. This knowledge gap highlights the importance of local biomechanical abnormalities in DFU development. The lack of uniform patient characteristics across studies represents a major limitation. Our review included only two studies that assessed the ankle-brachial index (ABI) [30,31], which is crucial for evaluating lower-limb vascular disease [54]. ABI assists clinicians in assessing vascular damage and determining whether amputation is required [55,56]. As ABI measurements are critical for clinical decision-making in DFU patients, their limited inclusion underscores the need for broader incorporation in future studies. Additional confounding factors may have contributed to clinical heterogeneity in this analysis. The limited sample sizes, incomplete methodological details, and short follow-up periods of several studies are further drawbacks. It is also important to note that one relevant study, Akram et al. (2024), was not included in this review because it was published after the predefined search cutoff date and was unavailable in indexed databases at the time of data extraction. Including it retrospectively would have introduced selection bias beyond the established inclusion framework. Likewise, future studies could further validate the efficacy of NPWT. The exclusive use of published and open-access material may lead to publication bias and an incomplete representation of evidence. Furthermore, funnel-plot analysis for complete ulcer closure revealed asymmetry, indicating potential publication bias. This suggests that smaller studies with non-significant findings may be underreported, possibly inflating the pooled effect estimate (OR = 2.19, 95% CI: 1.56-3.08; p < 0.0001). Therefore, although the meta-analysis demonstrates a statistically significant benefit, the observed advantage of NPWT should be interpreted cautiously, as the true magnitude of benefit might be smaller than observed due to selective publication of studies showing favourable outcomes. Future analyses should incorporate unpublished data and apply bias-correction methods to confirm result robustness.

Future

The field of NPWT is well positioned for a transformative future with a wide range of potential applications. Beyond wound healing, NPWT may also serve in targeted drug delivery, augmentation of stem-cell therapy [57], and integration with filtration devices [58]. The next generation of personalized care will likely feature adaptable smart dressings, remote monitoring, and wound imaging technologies. The relevance of NPWT extends to tissue engineering [59], chronic wounds, and post-surgical recovery [60], offering innovative options for improved outcomes across diverse medical settings. Well-executed research [61] demonstrated the use of a DIY negative-pressure device for skin grafts, showing effectiveness despite low cost. The ability for healthcare institutions to manufacture and install their own mobile devices [62] rather than invest in more expensive systems could significantly reduce financial burdens. Another study [63] employed a handmade NPWT system; although limited by methodological flaws, it still reported positive effects on wound healing. Combining NPWT with other treatment modalities has produced promising results. The combination of NPWT and Hyperbaric Oxygen Therapy (HBOT) shows effective synergy [64,65]; HBOT’s oxygen-rich environment enhances tissue oxygenation and angiogenesis, complementing the wound-healing properties of NPWT [66]. In addition, complex wound dressings [67-70] and growth factors [71,72] can be used alongside NPWT to promote tissue regeneration. For infected wounds, antibiotics may be administered concurrently to control pathogens and accelerate healing [73]. In complex cases, surgical interventions such as debridement or reconstructive surgery can be integrated with NPWT. Compression therapy [74] may also be utilized to facilitate venous return and reduce edema, thereby complementing NPWT. This multidisciplinary approach requires collaboration among healthcare professionals to deliver faster, more comprehensive wound healing and ultimately improve patients’ quality of life. In conclusion, the findings from this meta-analysis strengthen existing evidence supporting NPWT in DFU management. The pooled odds ratio for complete ulcer closure (OR = 2.19, 95% CI: 1.56-3.08; p < 0.0001) closely aligns with Liu Z et al. [19] (OR = 2.04, 95% CI: 1.48-2.82) and Chen L et al. [75] (OR = 3.60, 95% CI: 2.38-5.45; p < 0.001). For granulation tissue formation, our SMD = -1.34, 95% CI: -1.56 to -1.12; p < 0.0001 parallels Chen L et al. [75], who reported a mean difference of -8.95 days (95% CI: -10.26 to -7.64; p < 0.001), both confirming faster wound-bed preparation with NPWT. Safety outcomes were consistent across studies, with no significant difference in adverse events. Collectively, these robust and reproducible findings validate NPWT’s clinical efficacy and support its integration into future wound-care strategies, particularly within personalized, technology-assisted, and cost-effective treatment frameworks.

## Conclusions

In conclusion, this systematic review and meta-analysis demonstrate that NPWT is a potent, adaptable, and cost-effective strategy for the management of DFUs. NPWT significantly improves key outcomes such as ulcer closure, wound healing, granulation tissue formation, and reduction in amputation rates, as shown by multiple studies, thus supporting its use over standard treatment modalities. Measuring granulation tissue development may be more clinically informative than focusing solely on ulcer closure or ulcer size. Regarding safety, NPWT exhibits a comparable rate of adverse events to conventional therapies, and controlled clinical settings have reported no serious complications when standardized protocols are followed. However, future research is required in ambulatory care settings, where application may be more challenging. By expediting wound healing, NPWT can lead to lower overall treatment costs and improved healthcare resource utilization. Overall, this meta-analysis provides substantial evidence for the efficacy, safety, and cost-effectiveness of NPWT in DFU management. Continued research and interdisciplinary collaboration are essential to enhance protocols and extend the benefits of NPWT to diverse clinical environments.
